# Supplementary material for: Centennial response of Greenland’s three largest outlet glaciers
Source: Nat Commun. 2020 Nov 17;11:5718. doi: 10.1038/s41467-020-19580-5 (PMC7672108; doi:10.1038/s41467-020-19580-5)
Supplement: Supplementary file 1 — Supplementary Information [file 41467_2020_19580_MOESM1_ESM.pdf]

Supplementary Information

Centennial response of Greenland's three largest outlet glaciers

Shfaqat A. Khan*[1], Anders A. Bjørk[2], Jonathan L. Bamber[3], Mathieu Morlighem[4], Michael Bevis[5], Kurt H. Kjær[6], Jérémie Mouginot[7], Anja Løkkegaard[1], David M. Holland[8], Andy Aschwanden[9], Bao Zhang[10], Veit Helm[11], Niels J Korsgaard[12], William Colgan[12], Nicolaj K Larsen[6], Lin Liu[13], Karina Hansen[1], Valentina Barletta[1], Trine S Dahl-Jensen[1], Anne Sofie Søndergaard[14], Beata M. Csatho[15], Ingo Sasgen[11], Jason Box[12], Toni Schenk[15]

1. DTU Space, Technical University of Denmark, Denmark
2. University of Copenhagen, Denmark
3. Bristol Glaciology Centre, University of Bristol, UK.
4. Department of Earth System Science, University of California, Irvine, USA
5. School of Earth Sciences, Ohio State University, USA.
6. Globe Institute, University of Copenhagen, Denmark
7. Institut des Géosciences de l'Environnement, Université Grenoble Alpes, France
8. Center for global Sea Level Change, New York University Abu Dhabi, UAE
9. University of Alaska Fairbanks, USA
10. School of Geodesy and Geomatics, Wuhan University, China
11. Glaciology Section, Alfred Wegener Institute, Germany
12. Geological Survey of Denmark and Greenland, Denmark
13. Earth System Science Programme, The Chinese University of Hong Kong, Hong Kong
14. Dept. for Geoscience, Aarhus University, Denmark
15. Department of Geology, University at Buffalo, USA

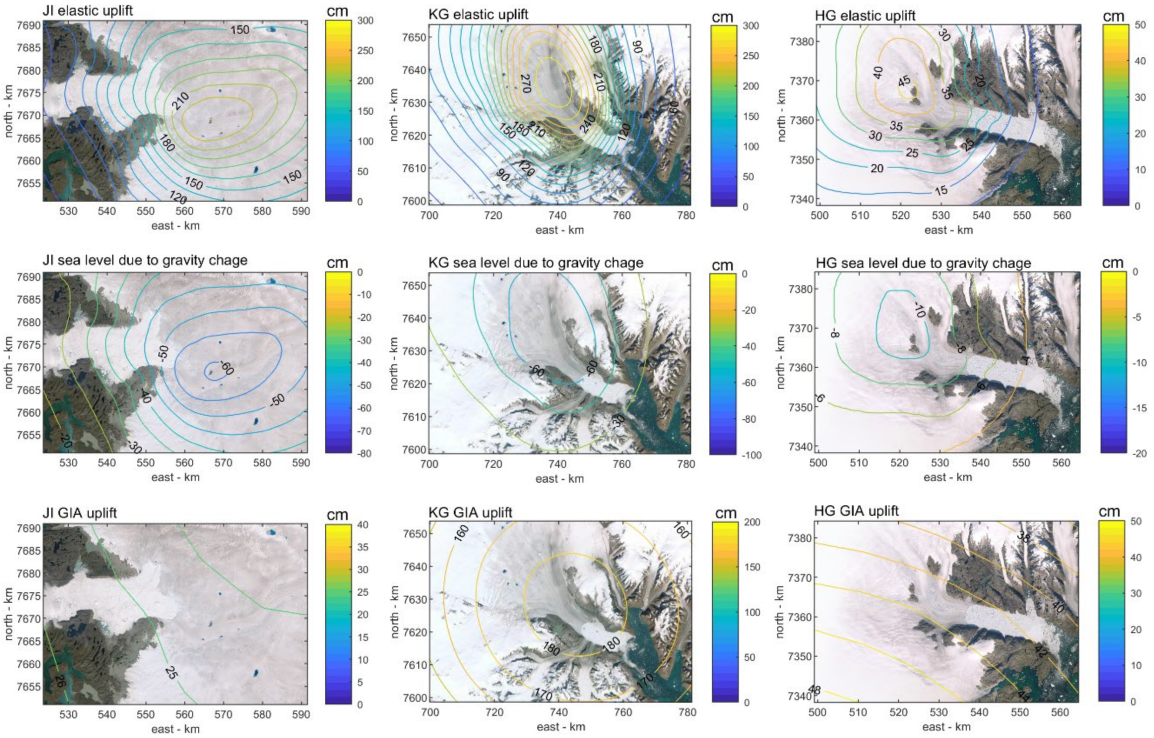

Supplementary Figure 1. Bedrock uplift and sea level change.

Accumulated elastic uplift, sea level change due to reduced gravity, and uplift due to glacial isostatic adjustment at Jakobshavn Isbræ (JI), Kangerlussuaq Glacier (KG) and Helheim Glacier (HG) during LIA-2012. Uplift of bedrock and sea level are provided in cm.

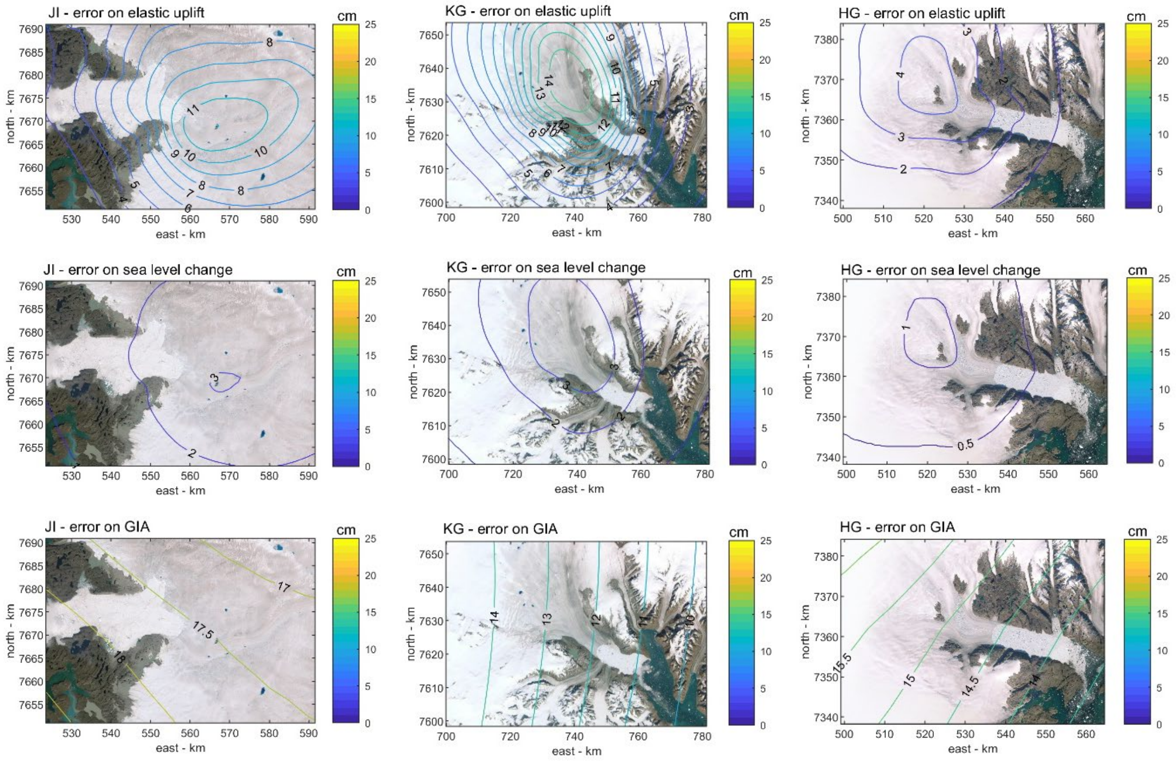

Supplementary Figure 2. Uncertainties.

Uncertainties related to each of the panel in Supplementary Fig. 1.

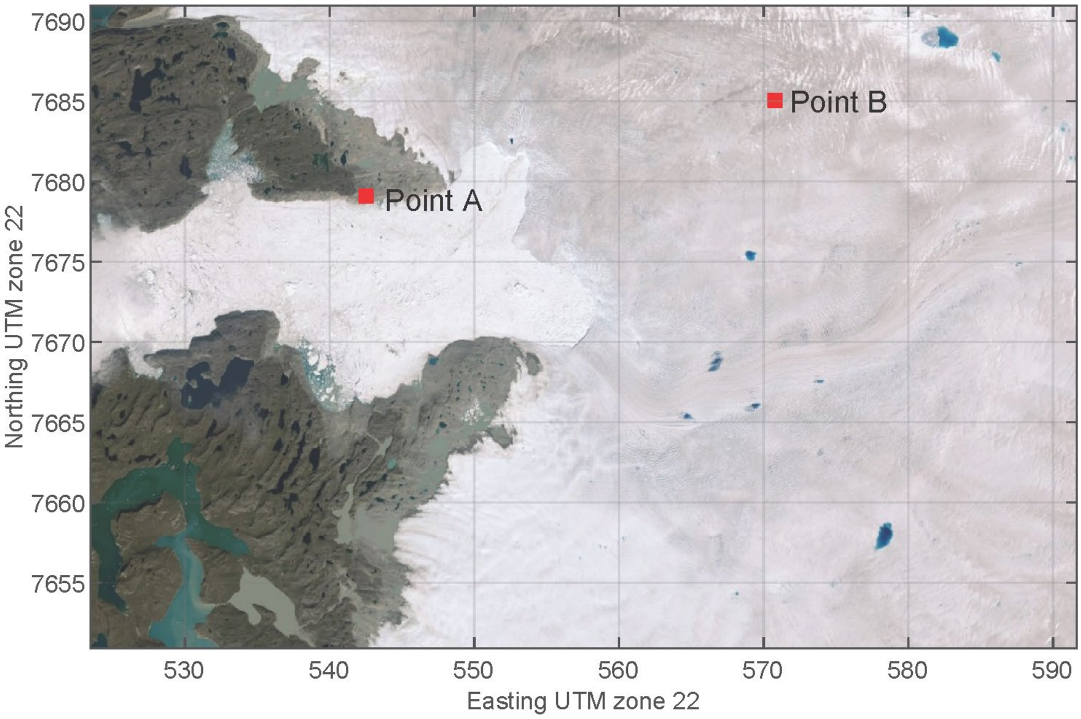

Supplementary Figure 3. Map of Jakobshavn.

Map of Jakobshavn Isbræ provided in UTM zone 22 coordinates in km. Time series of elevations at point A and B are shown in Supplementary Fig. 4.

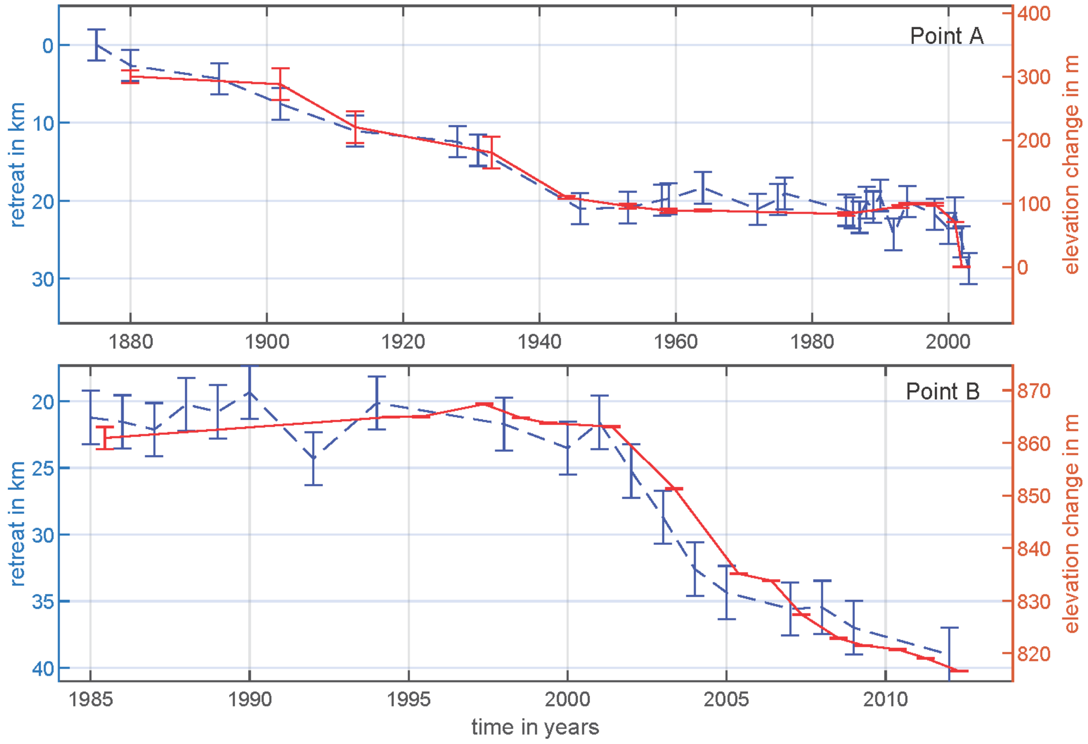

Supplementary Figure 4. Elevation change.

Time series of elevation change at point A and B in meter (right axis) and front retreat in km (left axis).

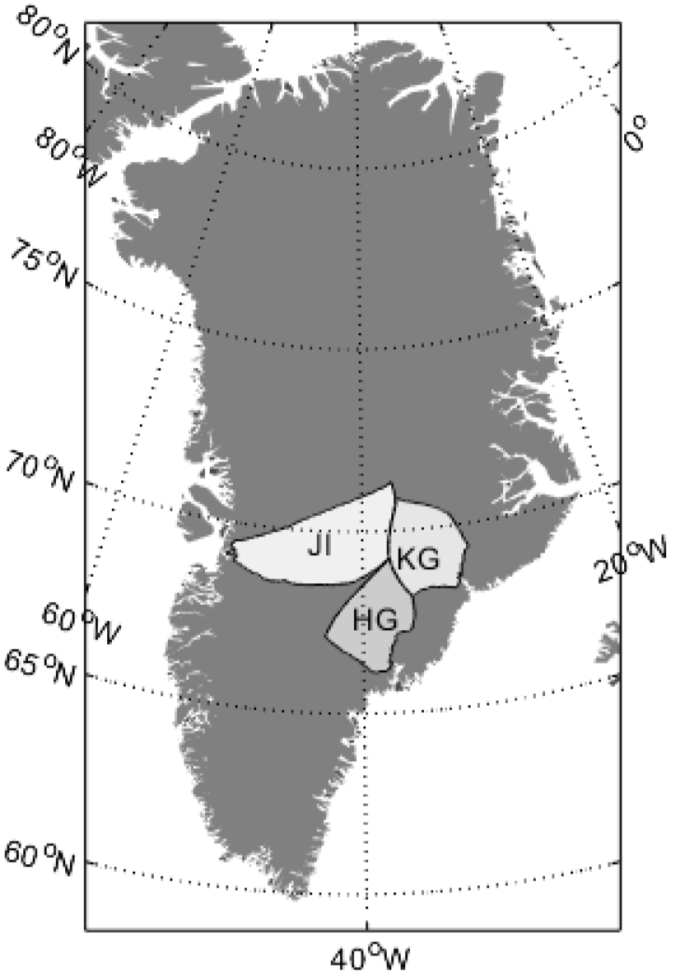

Supplementary Figure 5. Map of Greenland.

Map of Greenland and the drainage area of Jakobshavn Isbræ (JI), Kangerlussuaq Glacier(KG), and Helheim Glacier (HG).

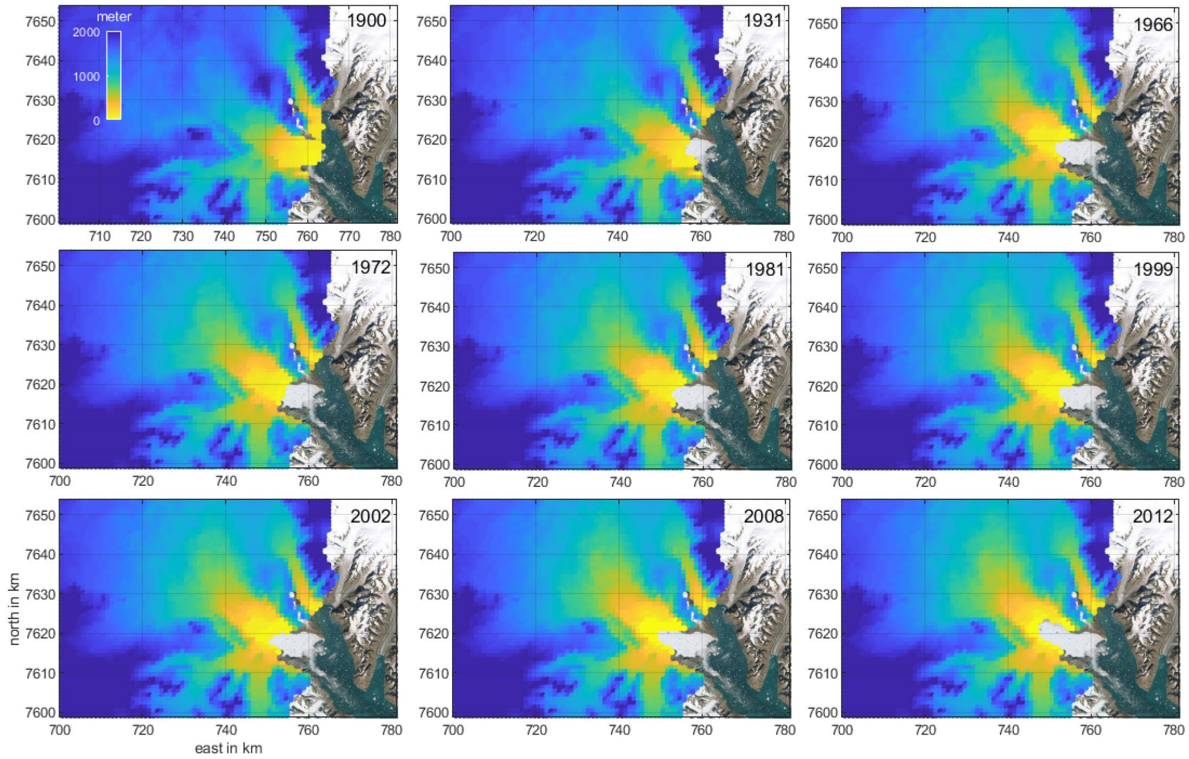

Supplementary Figure 6. Surface elevations.

Kangerlussuaq Glacier surface elevations in meter during 1880, 1931, 1966, 1972, 1981, 1999, 2002, 2008 and 2012.

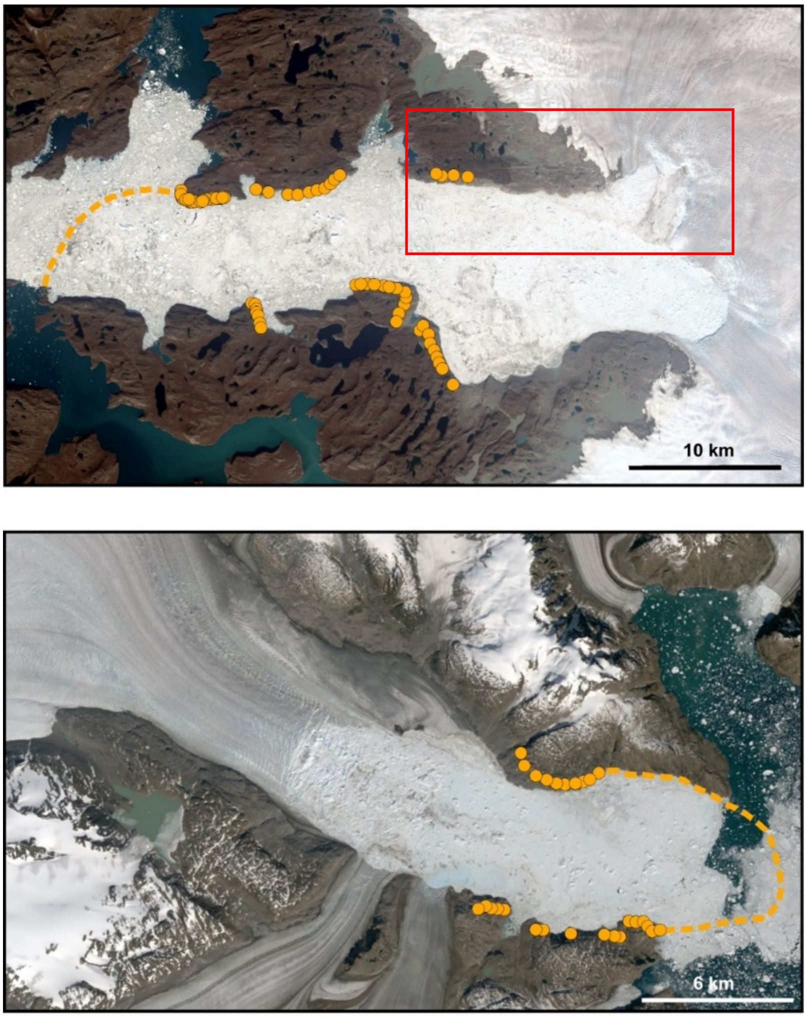

Supplementary Figure 7. Little Ice Age marginal positions.

The pre-1980s LIA marginal positions used to calculate total post-LIA mass loss. Hatched line shows the proposed LIA maximum position. The area marked by a red box is shown in Supplementary Fig. 8.

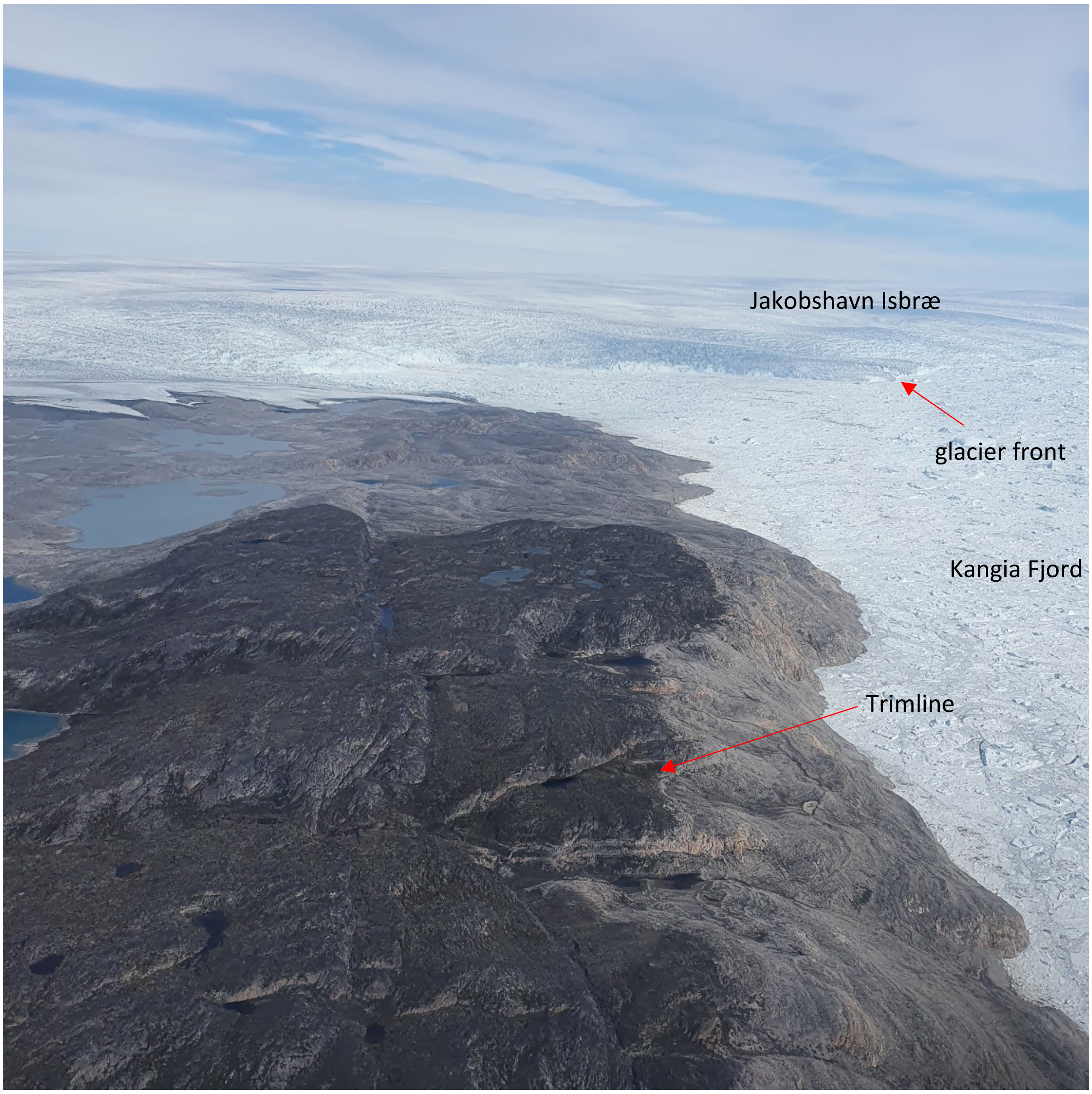

Supplementary Figure 8. Photo of Jakobshavn Isbræ.

Photo of the norther section of Kangia fjord next to Jakobshavn Isbræ acquired in July 2020.

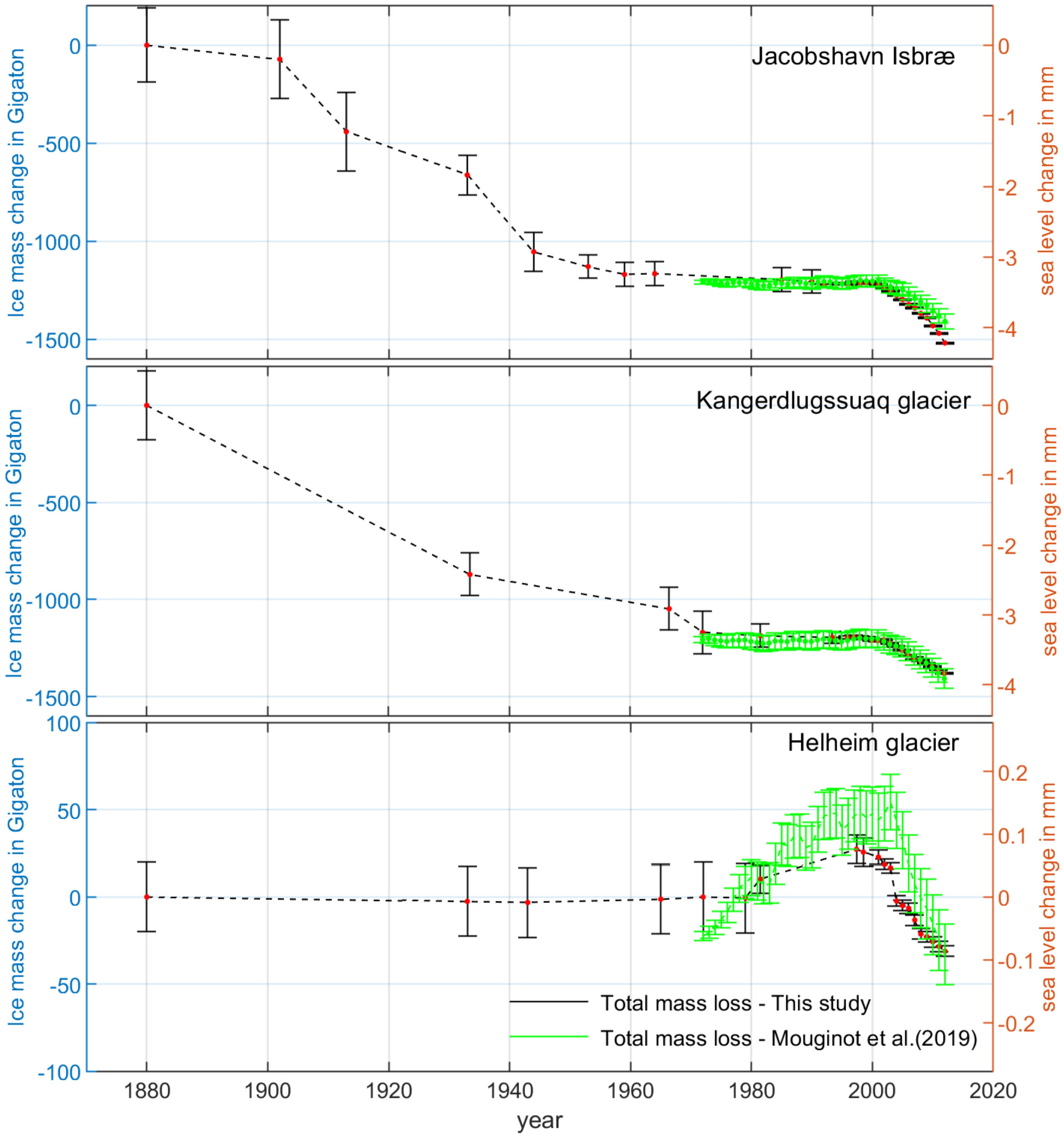

Supplementary Figure 9. Ice mass loss.

Time series of accumulated ice mass loss of Jakobshavn Isbræ, Kangerlussuaq Glacier and Helheim Glacier. Black curve denotes this study. Green curve denotes Mouginot et al. (2019)[15]. Vertical lines denote error bars.

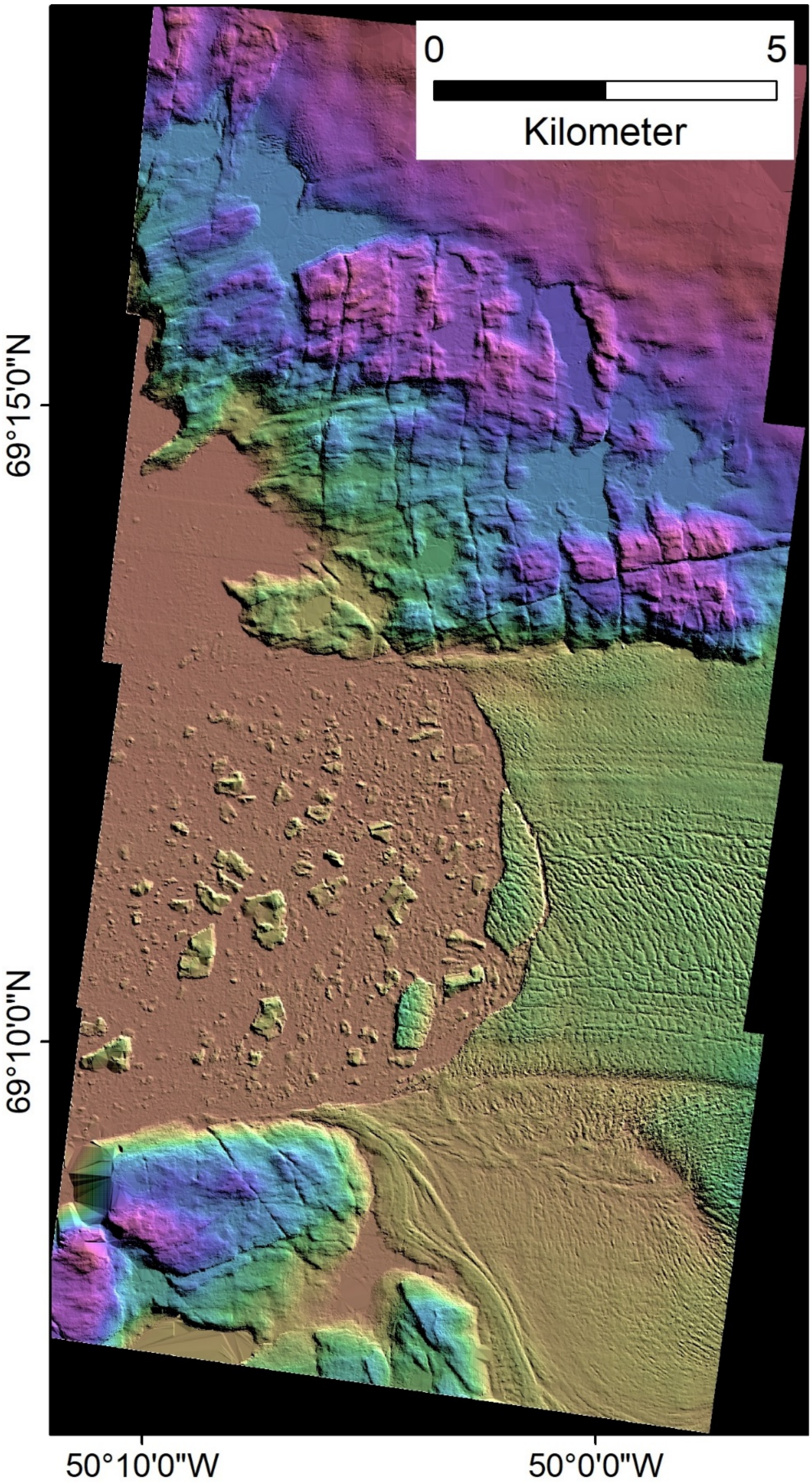

Supplementary Figure 10. Digital Elevation Model.

Hillshade of digital elevation model for the frontal portion of Jakobshavn Isbræ based on 1964 Aerial photographs.
