# Supplementary material for: Centennial response of Greenland’s three largest outlet glaciers
Source: Nat Commun. 2020 Nov 17;11:5718. doi: 10.1038/s41467-020-19580-5 (PMC7672108; doi:10.1038/s41467-020-19580-5)
Supplement: Supplementary file 3 — Description of Additional Supplementary Files [file 41467_2020_19580_MOESM3_ESM.pdf]

Description of Additional Supplementary Files

File Name: Supplementary Movie 1
Description: Annual front positions of Jakobshavn Isbræ and surface elevations in meter during 1875-2012.
