# Supplementary figures and images for: Centennial response of Greenland’s three largest outlet glaciers
Source: Nat Commun. 2020 Nov 17;11:5718. doi: 10.1038/s41467-020-19580-5 (PMC7672108; doi:10.1038/s41467-020-19580-5)

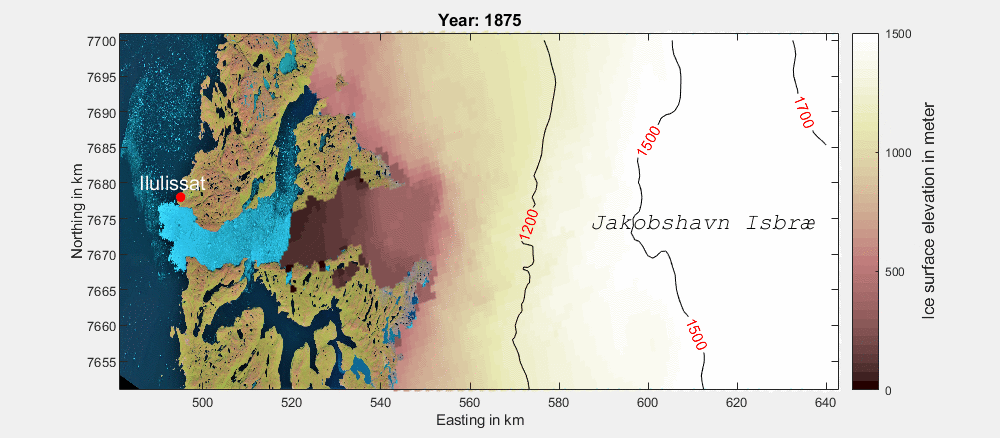

Supplement: Supplementary file 4 — Supplementary Movie 1 [file 41467_2020_19580_MOESM4_ESM.gif]
